# COVID-19, income and gender wage gap: Evidence from the China family panel studies (CFPS) 2014 to 2020

**DOI:** 10.3389/fpubh.2022.1066625

**Published:** 2022-11-25

**Authors:** Haojian Dui

**Affiliations:** School of Labor and Human Resources, Renmin University of China, Beijing, China

**Keywords:** COVID-19, income, gender wage gap, telecommuting, Generalized Difference-in-Differences

## Abstract

COVID-19 has a ubiquitous impact on human society and a significant impact on the labor market. This paper explores the impact of COVID-19 on income and its gender differences based on Generalized Difference-in-Differences using publicly available national micro-tracking survey data (CFPS 2014–2020) for the first time. The main findings are as follows: 1. COVID-19 significantly reduces incomes and affects men more; 2. Telecommuting mitigates income losses and is a significant factor contributing to the smaller impact on women; 3. There is educational heterogeneity in COVID-19 shock, with a significant negative impact on the income of those with lower education and a non-significant impact on those with higher education; 4. Men working in production and transportation, as well as female workers in commerce and services, will suffer the greatest loss of income; 5. For men, the older they are, the more they are affected by COVID-19, while the opposite is true for women; 6. Compared to urban residents, COVID-19 has a greater impact on rural residents. There are some policy implications: 1. the relationship between COVID-19 prevention measures and economic development should be carefully considered. 2. Telecommuting should be promoted during the COVID-19 pandemic. 3. The vulnerable groups should be protected.

## Introduction

As of August 28, 2022, the cumulative number of confirmed COVID-19 cases worldwide has exceeded 600 million^1^. The outbreak of COVID-19 has caused enormous health and economic losses worldwide, and its powerful contagion has forced governments to take strict prevention measures to safeguard people's lives. The epidemic itself and the accompanying prevention measures have profoundly changed the way we live and work, and the labor market has suffered a dramatic impact. Based on current developments, the epidemic will continue to spread around the world in the short term, and even if the epidemic disappears in the future, its far-reaching effects will be difficult to eliminate. Studies have shown that the epidemic has caused severe unemployment, loss of income, and mental health problems (1–4).

The gender wage gap has been a long-standing concern in academia, with important implications for individual development, family stability, economic growth, wealth disparity, and intergenerational mobility (5–7). Worryingly, the gender wage gap in China has been rapidly widening as the economic transition has progressed over the decades (8), it has created an obstacle to achieving a fair income distribution pattern and the strategic goal of common prosperity. How will the gender wage gap in China be affected by the dramatic changes in the way people live and work under the influence of COVID-19? Further research is needed.

The purpose of this paper is to explore the effect of COVID-19 on income and its gender differences. Using Generalized Difference-in-Differences (Generalized DID), baseline regressions were conducted to examine the effect of COVID-19 on incomes, and gender differences in the effect of COVID-19 were explored in subsamples and then I did a series of rigorous tests. On this basis, I analyzed the heterogeneous effects of COVID-19 at the educational, occupational, age, and rural-urban levels, and explored the mechanistic role of telecommuting.

The marginal contributions and implications of this paper are mainly in the following areas:

Firstly, according to Adams-Prassl et al. (9–13), it can be found that the impact of COVID-19 varies significantly across countries. China was the first country to be hit by COVID-19, and it has always adhered to the dynamic zero-COVID policy, and has implemented stricter epidemic prevention measures than most other countries. Therefore, the development and impact of COVID-19 in China is certainly different from that of other countries. Compared with Western countries, there are fewer studies on China's reality, especially the lack of empirical studies with nationally representative data, which is not conducive to the understanding of China's reality and the formulation of epidemic prevention measures and economic policies in the post-COVID-19 era. This study can help to understand the impact of COVID-19 in China and provide ideas for subsequent policy development.

Secondly, according to the World Bank, the female labor force participation rate of China was 62% in 2021, it was well above the world average of 46% and ranking among the world's leading economies^2^. Exploring how COVID-19 affects the gender wage gap in the context of persistent COVID-19 perturbations is crucial to safeguarding women's income and labor market equity in the post-COVID-19 era, as well as to the economic development of China after COVID-19.

Thirdly, the rapid spread of telecommuting during COVID-19 has profoundly changed the way people work and impacted the labor market landscape. According to the 49th “Statistical Report on Internet Development in China” released by China Internet Network Information Center (CNNIC), the number of online office users in China has reached 469 million by December 2021. And according to the research data of PwC's “2022 Global Workplace Survey (Mainland China),” employees in Mainland China have a strong desire to telecommute, and 95% of them want to implement telecommuting and hybrid mode in the future. So, even if COVID-19 ends in the future, it is expected that telecommuting will still be an important way of working. In this context, telecommuting as a gender-differentiated impact mechanism of COVID-19 can not only help us understand the reality of COVID-19 impact, but also provide ideas to alleviate the gender wage gap in the long run.

Finally, the data used in this study are authoritative and representative, and the methods are scientific and reasonable and have been rigorously tested to ensure the robustness of the results. At present, there is no unified view on the conclusions and mechanisms of the studies on these issues, so this paper can provide reliable evidence for subsequent studies.

This paper is organized as follows: The first part is the introduction, which introduces the background and main content of this study, and on this basis, the main marginal contributions and implications of this paper are explained; the second part is the literature review, which reviews the existing studies; the third part is the data and variables, which describes the data sources and specific variables set in this paper; in the fourth section, the empirical model and the methods used are presented, followed by the baseline regression and the presentation of the results. Then parallel trend tests, robustness tests, and placebo tests were performed; the fifth section provides further analysis, starting with a heterogeneity analysis of the effects of COVID-19 and its gender differences along the four dimensions: education, occupation, age, and urban/rural. Then, the mechanistic role of telecommuting experience is explored; the sixth section, Conclusions and Policy Implications, describes the main work of this paper and the findings based on the empirical results. Then, in the context of China's reality, suggestions are made to improve the overall income level and alleviate the gender wage gap in the post-COVID-19 era.

## Literature review

This paper explores the impact of COVID-19 from the perspective of income and further analyzes the impact of COVID-19 on the gender wage gap. There is a consensus in the literature that COVID-19 has a negative impact on income, but scholars still hold different views on how COVID-19 affects the gender wage gap. Brodeur et al. (13) shows that while men are usually more affected by macro shocks in previous studies of recessions, the current COVID-19 shock reveals many factors that are more detrimental to women. Adams-Prassl et al. (9) and Dang and Nguyen, (14) showed that women suffered more income loss due to COVID-19. However, Liang et al. (15) used data from a two-period follow-up survey in vocational high schools and found that COVID-19 had a greater negative impact on men's income. Gambau et al. (16) also found that COVID-19 makes men more vulnerable to poverty. It can be observed that most of the available studies are based on foreign contexts, and the number of studies based on China is relatively small. Therefore, this paper will provide new evidence on COVID-19 and the gender wage gap by using nationally representative data to empirically analyze Chinese reality.

What mechanism caused the different effects between genders in COVID-19? This question has also triggered extensive scholarly discussion. In the existing literature, scholars generally agree that women are more affected by COVID-19 due to the following factors: industries with more female workers are more affected by COVID-19; a greater proportion of women are working in temporary jobs; and because schools and childcare services are often forced to close during COVID-19, resulting in mothers sacrificing more energy to care for their children (9, 17, 18). Studies suggesting that men are more affected by COVID-19 have found that industries with a higher proportion of men are more affected by COVID-19 (15), or that women benefit more from telecommuting (19). As we can see, there is still no consensus on either gender differences or the mechanisms that influence them, and there is a paucity of studies based on Chinese reality, so further research on these issues is needed.

The explosion of COVID-19 passively accelerated the diffusion of telecommuting, which is the main difference between the labor market impact of COVID-19 and previous macro shocks, and therefore produces different impact mechanisms and effects. It has been found that workers who are able to telecommute are generally better paid than those who are not (20, 21), and that more educated groups are more likely to telecommute (21–23). In terms of gender differences, some scholars have found that men are more likely to telecommute (21, 23, 24), while others have noted that telecommuting is more beneficial to women (19, 22, 25). This paper analyzes the mechanistic role of telecommuting in the impact of the epidemic on the gender wage gap, in order to provide support for the different views on the effects of telecommuting in existing studies.

In addition to gender differences, there are some heterogeneities in other dimensions of COVID-19 shock to income. The findings on the effect of education are almost consistent, i.e., less educated groups are generally subject to larger negative shocks compared to the highly educated (26, 27). On the industry side, del Rio-Chanona et al. (28) analyzed the differences in the exposure of different industries to COVID-19, noting that transportation, manufacturing and mining, entertainment and restaurants, and tourism were subject to significant shock on the demand, supply, supply and demand sides, respectively. Albanesi and Kim (29) found that the severity of the impact of COVID-19 varied across occupations due to their flexibility and sociability, with occupations such as healthcare and services, which are difficult to telecommute and require high levels of proximity, being the most affected. This effect is also reflected in the gender wage gap, as there are significant differences in the gender ratio within occupations. In terms of age, most of the existing studies suggest that younger people are more affected by COVID-19 (18, 30). In contrast, Hoehn-Velasco et al. (31) showed that both the youngest and oldest groups of workers were severely affected, while Hoshi et al. (32) found that COVID-19 caused more unemployment and hence loss of income among older workers. It could be found that there is significant heterogeneity in the effects of COVID-19 shock on different groups, and the findings are not identical. This paper examines these heterogeneous effects and analyzes urban-rural heterogeneity in the context of China's dualistic economy.

By reviewing the available literature, it was found that the impact of COVID-19 on income and gender differences has attracted widespread attention, but studies on this issue still have the following shortcomings: First, there are few studies based on Chinese reality; second, many empirical studies use small-scale online surveys, local surveys, and other methods, and the samples are subject to selection bias and under-representation, and the unpublished data make it impossible to verify the results of the articles. Finally, there is no consensus on the effects of COVID-19 on income and gender differences in terms of conclusions and mechanisms, and more robust evidence is needed to support them.

## Data and variables

### Data

The individual micro-data used in this paper was obtained from the China Family Panel Studies (CFPS) for four periods from 2014 to 2020. The reasons for using CFPS data are as follows: First, the systematic probability sampling method used in CFPS ensures its nationwide representativeness; second, CFPS, as a large micro tracking survey data, can constitute panel data and has good properties; finally, CFPS2020 is the first publicly available micro-data in China that contains COVID-19-related variables and is nationally representative, which can provide sufficient data support for this study. In addition, the data of COVID-19 cases was obtained from the national and provincial health committees. Other provincial data were obtained from the China Statistical Yearbook for each year. The sample was limited to the working-age population (male: 16–60 years old; female: 16–55 years old), and the sample of Hubei Province was excluded to obtain unbalanced panel data^3^ with a sample size of 17,141 after cleaning missing and outliers.

### Variables

#### Dependent variable

The dependent variable in this paper is the monthly income of individuals (*income*_*i*_), and it is logarithmized in the empirical analysis. It can be further divided into pre-COVID-19 income (in 2014, 2016, 2018) and post-COVID-19 income (in 2020). Pre-COVID-19 income is calculated by dividing the “after-tax wage income from all jobs in the past 12 months” in the CFPS data by 12 to calculate the average monthly income.

Income after COVID-19 was calculated based on the above question and the CFPS questionnaire “How did your monthly income change in February and March 2020, when COVID-19 was most severe in the country,” and “By what percentage did your monthly income change compared to your regular monthly income? ”These two questions were calculated by taking into account the cumulative number of cases and new cases per month in each province, as well as the month in which the respondents were interviewed. Considering the real-world impact of COVID-19, it was assumed that the impact of new cases would last for 1 month and the degree of impact would be related to the number of cases.

The CFPS data for COVID-19-related issues is limited to February and March 2020, which is the period of concentrated outbreak of COVID-19 in China, and due to the inexperience in fighting the epidemic in the early stage of COVID-19, almost all regions are affected by the biggest impact during this period, so the impact during this period is taken as the baseline impact. The calculation of the specific impact on each individual in combination with the baseline impact takes into account the economic resilience of each region and the real impact on each individual, which makes the calculation results more realistic. The specific calculation process for post-COVID-19 income is as follows:

The coefficient for February and March 2020 will be set as 1. The formula for calculating the coefficient (*ceffect*_*pj*_) for each province affected from April to December is as in equation (1):


(1) $$\begin{array}{l} ceffect_{pj}=\frac{covid_{pj}}{MAX\left(covid_{pk}\right)},\;p\in \left[1,31\right],\;j\in \left[4,12\right],\;k\in \\ \;\left[2,12\right] \end{array}$$

Where, *covid*_*pj*_ is the number of new COVID-19 cases in region *p*, the *j*-th month. *MAX*(*covid*_*pk*_) is the maximum number of new COVID-19 cases in a single month from February to December in region *p*. Coefficient *ceffect*_*pj*_ is a value in the interval [0,1].

The percentage change in income for individual *i* in February and March 2020 compared to the regular months was calculated using the two questions about COVID-19 revenue in CFPS mentioned above. The change in income of individual *i* in the *j*-th month *incomev*_*ij*_ is calculated by equation (2):


(2) $$\begin{array}{l} incomev_{ij}=ceffect_{pj}\times incomev_{i0} \end{array}$$

Let *income*20_*i*_ be the total income of individual *i* in CFPS2020 in the 12 months before the interview, *rinc*_*i*_ be the regular average monthly income^4^, and *c*_*i*_ be the month of the interview^5^, so that the equation (3) was obtained:


(3) $$\begin{array}{l} {income20}_{i}=\left(14-c_{i}\right)\times rinc_{i}+2rinc_{i}\times \left(incomev_{i0}+1\right)\\ \;+\overset{c_{i}-1}{\underset{j=4}{\sum }}rinc_{i}\times \left(incomev_{ij}+1\right) \end{array}$$

The first term on the right-hand side of equation (3) represents the total income of individual *i* in the regular month before COVID-19, the second term is the total income in February and March 2020, and the third term is the total income from April to the month before the interview.

Finally, the average monthly income of the individual *i* after COVID-19 *covidinc*_*i*_ can be calculated by equation (3) as:


(4) $$\begin{array}{l} \;covidinc_{i}={income20}_{i}\times \\ \left[1-\frac{\left(14-c_{i}\right)}{12+2incomev_{i0}+\sum_{j=4}^{c_{i}-1}\left(incomev_{ij}\right)}\right]\times \frac{1}{c_{i}-2} \end{array}$$

#### Independent variable

The independent variable in this paper is the shock of COVID-19 *covidcm*_*i*_. Considering the size of the population in each region and the different extent and duration of exposure to COVID-19 for each individual, I calculated COVID-19 shock using the average monthly exposure of individual *i* after the national COVID-19 outbreak. The specific calculation method is as in equation (5):


(5) $$\begin{array}{l} covidcm_{i}=\frac{covidc_{i}}{c_{i}-2} \end{array}$$

Where, *covidc*_*i*_ is the cumulative number of confirmed cases per 10,000 people in the province where individual *i* is located up to the month of interview. *c*_*i*_ is the month of interview for individual *i*.

#### Control variables

Based on the literature and available data, this paper selects control variables at three levels: individual characteristics, occupational characteristics, and regional characteristics, as shown in Table 1.

**Table 1:** Description of control variables.

**Characteristics dimension**	**Variables**	**Variables explanation**
Individual	Gender	Male = 1, female = 0
	Age	actual age at the time of interview (in years)
	Age squared/100	Age squared divided by 100
	Marital status	Married = 1, unmarried/single = 0
	Hukou status	Non-agricultural/residential hukou = 1, agricultural hukou = 0
	Political appearance	CPC member = 1, non-member of CPC = 0
	Years of education	Number of academic years a person completed in a formal program
	Self-rated health	Unhealthy = 1, fair = 2, good = 3, very good = 4, excellent = 5
Occupational	Occupational category	Current top job/most recently completed job occupation code①
Regional	Urban/rural classification of residence	Urban = 1, rural = 0

Gender was not used as a control variable because individual fixed effects were already controlled in the baseline regression.

①Due to the limitation of data, occupations are divided into following 9 types: Persons in charge of state organs, party and mass organizations, enterprises, and institutions = 1; professional and technical personnel = 2; clerks and related personnel = 3; business and service personnel = 4; production personnel in agriculture, forestry, animal husbandry, fishery, and water conservancy = 5; production and transportation equipment operators and related personnel = 6; military personnel = 7; non-professionals = 8; other employees who are inconvenient to classify = 9.

Table 2 reports the results of descriptive statistics for the main variables by sample years.

**Table 2:** Descriptive statistics of main variables.

**Variables**	**Year of the samples**			
	**2014**	**2016**	**2018**	**2020**
Income (logarithmic)	6.015 (3.151)	6.201 (3.197)	7.312 (2.327)	8.010 (1.033)
COVID-19 shock	/	/	/	0.021 (0.015)
Gender	0.629 (0.483)	0.609 (0.488)	0.611 (0.488)	0.608 (0.488)
Age	35.600 (9.222)	36.690 (9.638)	37.710 (10.020)	39.450 (9.984)
Age squared/100	13.520 (6.641)	14.390 (7.210)	15.230 (7.717)	16.560 (8.076)
Marital status	0.824 (0.381)	0.815 (0.389)	0.797 (0.402)	0.810 (0.393)
Hukou status	0.392 (0.488)	0.387 (0.487)	0.384 (0.487)	0.389 (0.488)
Political appearance	0.112 (0.316)	0.129 (0.335)	0.145 (0.353)	0.153 (0.360)
Years of education	10.480 (3.809)	10.850 (3.885)	11.250 (3.906)	11.150 (3.928)
Self-rated health	3.403 (1.064)	3.280 (1.075)	3.240 (1.046)	3.262 (1.044)
Occupational category	4.454 (1.740)	4.306 (1.923)	4.160 (1.762)	4.195 (1.822)
Urban/rural classification of residence	0.590 (0.492)	0.624 (0.484)	0.658 (0.474)	0.652 (0.476)
Observations	3,097	4,253	4,399	5,392

The dates in the table are the sample means, and the standard deviations are in parentheses.

## Empirical analysis

### Econometric model

The sudden outbreak of COVID-19 is unpredictable and thus can be viewed as a completely exogenous shock, and studied as a random natural experiment using the DID method. And the impact of COVID-19 is widespread, almost all regions have been affected by COVID-19, but there are differences in the specific extent. Based on these characteristics, as well as the characteristics of the data I used, this study could not use the conventional DID method to clearly distinguish the treatment group from the control group. In view of this, this paper refers to the methods of Nunn and Qian (33, 34) and other literatures, uses the Generalized DID method to set the econometric model with the COVID-19 shock as a continuous processing variable, and compares the impact of COVID-19. The income changes of different individuals before and after the shock, this estimation strategy can obtain more information from the existing data to make more accurate estimates. In addition, the model controls for individual fixed effects and time fixed effects to remove the influence of factors such as individual time-invariant characteristics and other time-varying macro shocks.

The econometric model used in this paper is shown in equation (6):


(6) $$\begin{array}{l} \ln\;income_{it}=\beta_{0}+\beta_{1}covidcm_{i}{*time}_{t}+\beta_{2}X_{it}+\delta_{i}+\eta_{t}+\varepsilon_{it} \end{array}$$

Where, ln *income*_*it*_ is the logarithm of individual *i*'s monthly income in period *t*. *covidcm*_*i*_ is the COVID-19 shock. *time*_*t*_ is the time dummy variable, assigned as 1 in 2020 and 0 in previous years. *X*_*it*_ is the set of control variables, including individual, occupational, and regional characteristics. δ_*i*_ and η_*t*_ are the individual and time fixed effects, respectively. ε_*it*_ is the error term. β_0_ is the constant term, β_1_ is the coefficient of the interaction term between the COVID-19 shock and the time dummy, β_2_ is the coefficient of the control variables. β_1_ is the coefficient of interest in this study, which estimates the effect of COVID-19 on income.

### Baseline regression

In this paper, I first estimated the equation (6) using a two-way fixed effects model. Considering that the development of epidemic prevention measures, the release of case information, and the classification of risk areas during the COVID-19 outbreak were mainly at the district and county levels, the samples at the district and county levels were strongly correlated. Therefore, the model is estimated using the robust standard errors of clustering at the district and county levels to obtain more robust estimation results.

Table 3 presents the results of equation (6) using the full sample to estimate the average effect of the COVID-19 shock on income. The results in column (4) of the table indicate that, on average, after controlling for control variables of individual, occupational, and regional characteristics, a one-unit increase in the COVID-19 shock (i.e., an increase of 1 cumulative confirmed case per 10,000 persons per month) is associated with a significant decrease in personal income of about 11.16 percentage points^6^.

**Table 3:** Effect of COVID-19 on income (whole sample).

	**(1)**	**(2)**	**(3)**	**(4)**
COVID-19 shock	−11.26^***^	−11.17^***^	−11.07^***^	−11.16^***^
	(2.913)	(2.906)	(2.913)	(2.923)
Individual characteristics	NO	YES	YES	YES
Occupational characteristics	NO	NO	YES	YES
Regional characteristics	NO	NO	NO	YES
Observations	17,141	17,141	17,141	17,141
	0.549	0.550	0.550	0.550

*** p < 0.01, **p < 0.05, *p < 0.1. Robust standard errors for clustering at the district and county levels are in parentheses. All estimates control for individual and time fixed effects.

In addition to exploring the average effect of COVID-19 on income, the more important objective of this paper is to investigate the effect of COVID-19 on the gender wage gap. Therefore, the sample is divided by gender based on the full sample regression and estimated again using the same model and method. Table 4 shows the results of the estimating equation (6) for the gender-segregated sample. The results show that after controlling for the control variables of individual, occupational, and regional characteristics, when the COVID-19 shock increases by one unit, the income of the male and female samples drop significantly by about 13.52 and 7.6 percentage points, respectively, indicating that the COVID-19 shock causes greater income loss for males than for females. The results of different effects between males and females partly support the conclusions of Liang et al. (15), but are opposite to Adams-Prassl et al. (9) and Dang and Nguyen, (14).

**Table 4:** Effect of COVID-19 on income (by gender).

	**Male**				**Female**			
	**(1)**	**(2)**	**(3)**	**(4)**	**(1)**	**(2)**	**(3)**	**(4)**
COVID-19 shock	−13.73^***^	−13.77^***^	−13.47^***^	−13.52^***^	−7.653^**^	−7.502^**^	−7.497^**^	−7.600^**^
	(3.309)	(3.293)	(3.329)	(3.337)	(3.663)	(3.677)	(3.678)	(3.669)
Individual characteristics	NO	YES	YES	YES	NO	YES	YES	YES
Occupational characteristics	NO	NO	YES	YES	NO	NO	YES	YES
Regional characteristics	NO	NO	NO	YES	NO	NO	NO	YES
Observations	10,506	10,506	10,506	10,506	6,635	6,635	6,635	6,635
	0.530	0.531	0.532	0.533	0.581	0.583	0.583	0.583

*** p < 0.01,

** p < 0.05, *p < 0.1. Robust standard errors for clustering at the district and county levels are in parentheses. All estimates control for individual and time fixed effects.

### Parallel trend test

Satisfying the parallel trend assumption is a prerequisite for using DID, which requires that there is no significant difference in the trend between the treatment and control groups prior to the shock in order to ensure that the model estimates the true treatment effect. In this paper, this means that if COVID-19 did not occur, there is no significant difference in income between individuals potentially affected by COVID-19 to different degrees, i.e., it means that the likelihood or severity of an individual's exposure to a COVID-19 shock is not correlated with the individual's time-varying factors.

In this paper, the event study method is used to test for parallel trends, and the model used is in equation (7):


(7) $$\begin{array}{l} \ln\;income_{it}=\beta_{0}+\overset{k=2020}{\underset{k=2014}{\sum }}\beta_{1}covidcm_{i}{*year}_{k}+\beta_{2}X_{it}\\ \;+\delta_{i}+\eta_{t}+\varepsilon_{it} \end{array}$$

Where, *year*_*k*_ is a dummy variable for whether the sample is taken from year *k*. If yes, it is taken as 1, otherwise it is taken as 0. Other variables are the same as in equation (6).

Samples from a period prior to the occurrence of COVID-19, i.e., 2018, were used as controls. β_1_ is the coefficient of the difference in income of individuals in each year's sample who are affected by COVID-19 to different degrees. Since all individuals prior to COVID-19 were not affected by COVID-19, the estimation here applies the counterfactual idea of assuming that the individual was exposed to the same COVID-19 shock as in 2020 and using this to explore income differences among individuals potentially affected by COVID-19 to different degrees.

**Figure 1:**
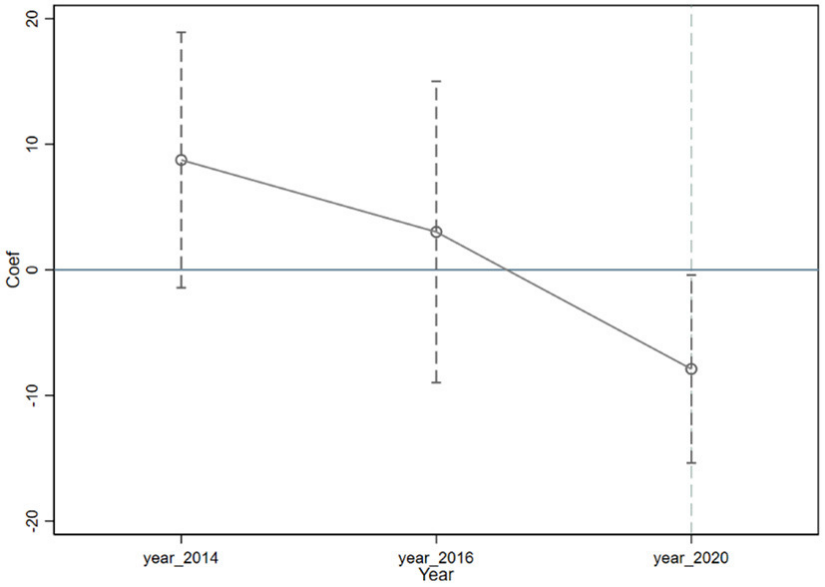
Parallel trend test graph. The hollow dots in the figure are the values of the coefficient β_1_ obtained by estimating equation (7), and the dotted lines indicate the 99% confidence interval. The estimation method uses a two-way fixed effects model and robust standard errors for clustering at the district and county levels.

Figure 1 is a parallel trend test diagram obtained by estimating equation (7). From the estimation results of the 2014 and 2016 samples, it can be seen that all estimates before the occurrence of COVID-19 are insignificant, indicating that overall the potential COVID-19 severity does not significantly affect individual income when COVID-19 does not occur, satisfying the parallel trend assumption and supporting the identification hypothesis of Generalized DID in this paper.

From the estimation results of the 2020 sample in Figure 1, the real COVID-19 shock has a significant negative correlation with individual income, i.e., the more severe the real COVID-19 shock is to individuals, the more their income decreases, which is consistent with the findings of the baseline regression.

### Robustness tests

On the basis of passing the parallel trend test, this paper will continue to conduct rigorous robustness tests from the following three perspectives to further verify the robustness of the results of this study.

#### Reconsideration of time trends

The baseline estimation model passed the parallel trend test, but it can be seen from Figure 1 that the estimated coefficients show a similar downward trend overall. Angrist and Pischke (35) suggest that when this situation happens, the estimation results are considered robust and convincing if a region-linked time trend term is added to the original DID model and the conclusions are consistent with the baseline regression. This is because the time fixed effects in the original model already control for common shocks at the time level between regions, and the time trend term in the new model takes into account the possibility of different time trends between regions before treatment, making the estimation results extremely robust.

Model (8) is constructed by adding the interaction term between the regional dummy variable γ_*c*_ and the time trend term *tyear*_*t*_ based on model (6):


(8) $$\begin{array}{l} \ln\;income_{it}=\beta_{0}+\beta_{1}covidcm_{i}{*time}_{t}+\beta_{2}X_{it}+{\beta_{3}\gamma }_{c}*tyear_{t}\\ \;+\delta_{i}+\eta_{t}+\varepsilon_{it} \end{array}$$

Table 5 shows the estimation results of model (8), using the same estimation method as in the baseline estimation.

**Table 5:** Robustness test (adding the time trend term).

	**Whole sample**	**Male**	**Female**
COVID-19 shock	−11.44^***^	−13.77^***^	−7.949^**^
	(2.984)	(3.374)	(3.771)
Time trend term	YES	YES	YES
Observations	17,141	10,506	6,635
	0.550	0.533	0.583

*** p < 0.01,

** p < 0.05, *p < 0.1. Robust standard errors for clustering at the district and county levels are in parentheses. All estimates control for individual and time fixed effects, as well as control variables of individual, occupational, and regional characteristics.

The results in Table 5 show that the estimation results are still significant after the inclusion of the region-linked time trend term, and the estimated coefficients are generally consistent with the baseline regression results. This indicates that the impact of COVID-19 on individual income is almost unaffected by the time trend difference between regions, which validates the robustness of the estimation results.

#### Changing the metric of COVID-19 shock

The year 2020 marks the beginning of the COVID-19 outbreak, and because COVID-19 was not well-understood and epidemic prevention measures were not well-developed, the presence of a single case in an area can often have an impact on the lives and work of residents. In this case, the presence of cases may have a greater impact on individual income than the number of cases. Therefore, the number of months that an individual was affected by COVID-19^7^ was considered as a proxy for the COVID-19 shock in the model (6) and re-estimated using the same estimation method as in the baseline regression, and the results are reported in Table 6.

**Table 6:** Robustness test (changing the metric of COVID-19 shock).

	**Whole sample**	**Male**	**Female**
COVID-19 shock	−0.0614^**^	−0.0633^*^	−0.0580^*^
	(0.0286)	(0.0338)	(0.0349)
Observations	17,141	10,506	6,635
	0.550	0.532	0.583

***p < 0.01,

** p < 0.05,

* p < 0.1. Robust standard errors for clustering at the district and county levels are in parentheses. All estimates control for individual and time fixed effects, as well as control variables of individual, occupational, and regional characteristics.

The results in Table 6 show that, COVID-19 shock has a significant negative effect on income overall, and the negative shock is greater for men than for women. This is fully consistent with the results obtained from the baseline regression, which further validates the robustness of the estimation results.

#### Changing the estimation strategy

In the above, the Generalized DID used in the baseline regression has passed the parallel trend test and undergone sufficient robustness tests to demonstrate the robustness of the results. However, given the complex steps involved in the construction of the post-COVID-19 income variables, there are inevitable errors between them and the true values. In order to further verify the robustness of the findings, the income change variable after COVID-19 *incomev*_*i*0_ is constructed as the dependent variable using the original data from the CFPS2020 questionnaire, and the model (9) is estimated using the OLS method as a robustness test.


(9) $$\begin{array}{l} incomev_{i0}=\beta_{0}+\beta_{1}{covid331}_{i}+\beta_{2}X_{i}+\varepsilon_{i} \end{array}$$

The dependent variable *incomev*_*i*0_ is the proportion of income change of individual *i* in February and March 2020 compared with the regular month, calculated from the direction and proportion of income change of respondents in February and March 2020 in the questionnaire. *covid*331_*i*_ is the cumulative number of cases in individual *i*'s province up to March 31, 2020 divided by 100. *X*_*i*_ is the set of control variables. ε_*i*_ is the error term. Since the OLS estimation of equation (9) uses cross-sectional data, which does not have the advantage of panel data, the possibility of endogeneity problems caused by omitted variables increases. Therefore, ethnicity^8^, regional GDP per capita, and other factors are controlled for in addition to the original control variables.

The results in Table 7 show that even with different estimation methods and variable settings, the results are still consistent with the baseline regression, i.e., the COVID-19 shock has a significant negative effect on income, and men are more affected than women, which fully validates the robustness of the results.

**Table 7:** Robustness test (OLS method).

	**Whole sample**	**Male**	**Female**
COVID-19 shock	−0.688^***^	−0.719^***^	−0.666^*^
	(0.205)	(0.241)	(0.363)
Observations	5,391	3,277	2,114
	0.132	0.173	0.085

*** p < 0.01,

**p < 0.05,

* p < 0.1. Robust standard errors for clustering at the district and county levels are in parentheses. All estimates control for individual and time fixed effects, as well as control variables of individual, occupational, and regional characteristics.

### Placebo test

To verify that the results are not due to other policies or unobservable factors, this paper uses a placebo test to further confirm the robustness of the results. In this paper, the effect of COVID-19 differs for each province in each month and can be viewed as each individual receiving a different intensity of treatment. Therefore, a placebo test can be conducted by randomly assigning the COVID-19 effects to each individual.

The COVID-19 effects were randomly assigned among individuals to generate a pseudo-COVID-19 effect variable *vcovid*_*i*_. Then, equation (10) was regressed using the same method as the baseline regression. In total, 500 random assignments and regressions were repeated in this paper.


(10) $$\begin{array}{l} \ln\;income_{it}=\beta_{0}+\beta_{1}vcovid_{i}{*time}_{t}+\beta_{2}X_{it}+\delta_{i}+\eta_{t}\\ \;+\varepsilon_{it} \end{array}$$

**Figure 2:**
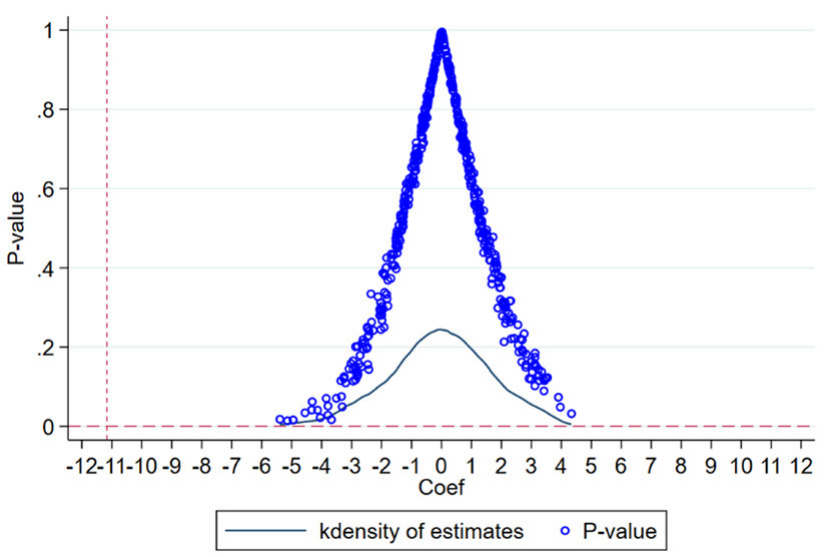
Placebo test. The X-axis represents the estimated value of coefficient β_1 in equation (9). The curve is the kernel density distribution, and the dots are the *p*-values. The intersection of the two dashed lines is the result of the baseline regression.

Figure 2 shows the results of the placebo test, and the result of the baseline regression is added for comparison. The estimated coefficients of the placebo test are concentrated around 0, with a normal distribution. The estimated result of the baseline regression (−11.16182, 0.00015) is located in the lower left corner of the axis, which is significantly different from the placebo test. The *p*-value of most of the estimates in the placebo test is bigger than 0.1, i.e., not significant at the 10% level. The *p*-value of the baseline regression results was < 0.001 and it was significant at the 1% level. In conclusion, the results of the placebo test demonstrate that the results of the baseline regression are hardly likely to be obtained by chance and are highly unlikely to be influenced by other policies or unobservable factors, further testing the robustness of the results.

## Further analysis

### Heterogeneous analysis

To further investigate the differences in the effects of COVID-19 shock on different groups, heterogeneous analyses were conducted separately from the perspectives of education, occupation, age, and urban/rural areas.

#### Education

To investigate the differences in the effects of COVID-19 shock across education-level groups, the sample was divided into those with less than high school education (11 years of education and below) and those with high school education and above (12 years of education and above). The same method as the baseline regression was used to regress equation (6), and the results are reported in Table 8.

**Table 8:** Heterogeneous analysis of education.

	**Less than high school education**			**High School education or above**		
	**Whole sample**	**Male**	**Female**	**Whole sample**	**Male**	**Female**
COVID-19 shock	−17.09^***^	−19.67^***^	−13.83^*^	−4.090	−6.098	−1.134
	(5.510)	(5.982)	(7.342)	(2.530)	(3.715)	(3.651)
Observations	8,263	5,458	2,805	8,878	5,048	3,830
	0.530	0.505	0.573	0.544	0.548	0.544

*** p < 0.01,

**p < 0.05,

* p < 0.1. Robust standard errors for clustering at the district and county levels are in parentheses. All estimates control for individual and time fixed effects, as well as control variables of individual, occupational, and regional characteristics. If I divide the results by tertiary education, we can get the same conclusion, with only a slight difference in coefficients, which is not shown due to space limitation.

The results in Table 8 show that COVID-19 has a significant negative impact on the income of those with low education, and it is still more affected by males. The negative impact of COVID-19 on those with high school education or above was not significant.

#### Occupation

In order to investigate the differences in the effects of COVID-19 shock across occupational groups, the sample was divided by occupational category (excluding military, unemployed, and other practitioners who were inconvenient to classify), and equation (6) was regressed using the same method as the baseline regression, and the results are reported in Table 9.

**Table 9:** Heterogeneous analysis of occupations.

	**Production and transportation workers**			**Person in charge**			**Commercial and service workers**		
	**Whole sample**	**Male**	**Female**	**Whole sample**	**Male**	**Female**	**Whole sample**	**Male**	**Female**
COVID-19 shock	−18.14^***^	−20.75^***^	−8.794	−15.51^**^	−18.76^*^	−8.191	−10.93^**^	−14.19^**^	−9.656^*^
	(4.608)	(5.183)	(8.746)	(7.403)	(10.40)	(12.72)	(4.535)	(6.726)	(5.607)
Observations	6,711	5,356	1,355	1,105	789	316	3,758	1,483	2,275
	0.506	0.499	0.541	0.607	0.568	0.703	0.568	0.563	0.572

*** p < 0.01,

** p < 0.05,

* p < 0.1. Robust standard errors for clustering at the district and county levels are in parentheses. All estimates control for individual and time fixed effects, as well as control variables of individual, and regional characteristics. The significance levels of the estimation results for all other occupations were above 10%, which are not shown due to space limitation.

Based on Table 9, the three occupational groups most severely affected by COVID-19 were: operators of production and transportation equipment and related workers; persons in charge of state organs, party organizations, enterprises, and institutions; and workers in commercial and service industries. Among the above occupations, the negative impact on men is greater than that on women. For men, production and transportation work were most affected by COVID-19. For women, workers in the commercial and service sectors suffered the greatest income loss.

#### Age

To investigate the differences in the effects of COVID-19 by age groups, the sample was divided into three parts: 35 years old and younger, 36–49 years old, and 50 years old and older, and equation (6) was regressed using the same method as the baseline regression, and the results are reported in Table 10.

**Table 10:** Heterogeneous analysis of age.

	**35 years old and younger**			**36–49 years old**			**50 years old and older**		
	**Whole sample**	**Male**	**Female**	**Whole sample**	**Male**	**Female**	**Whole sample**	**Male**	**Female**
COVID-19 shock	−7.748^**^	−6.263	−10.20^***^	−12.09^***^	−16.78^***^	−5.278	−15.99^***^	−18.95^***^	−4.881
	(3.316)	(5.077)	(3.901)	(4.406)	(5.009)	(6.173)	(5.631)	(5.594)	(9.935)
Observations	6,585	3,729	2,856	6,862	3,950	2,912	3,694	2,827	867
	0.537	0.517	0.568	0.559	0.541	0.589	0.545	0.529	0.600

Note:

*** p < 0.01,

** p < 0.05, *p < 0.1. Robust standard errors for clustering at the district and county levels are in parentheses. All estimates control for individual and time fixed effects, as well as control variables of individual, occupational, and regional characteristics.

The results in Table 10 show that the negative impact of COVID-19 on income increases with age for males and decreases for females. The loss of income due to COVID-19 was significantly greater for women than for men in the age group of 35 and below, while men were more affected in the age group of 36 and above. Overall, older age groups were more affected by COVID-19.

#### Urban/rural

To investigate the differences in the effects of COVID-19 by urban-rural differences, I divided the sample into rural and urban samples and regressed the equation (6) using the same method as the baseline regression, and the results are reported in Table 11.

**Table 11:** Heterogeneous analysis of urban-rural.

	**Rural**			**Urban**		
	**Whole sample**	**Male**	**Female**	**Whole sample**	**Male**	**Female**
COVID-19 shock	−15.26^**^	−17.71^**^	−13.85^*^	−3.987	−6.342^*^	−0.831
	(6.478)	(7.700)	(7.814)	(3.014)	(3.433)	(4.016)
Observations	5,876	3,990	1,886	11,265	6,516	4,749
	0.562	0.535	0.621	0.531	0.520	0.550

***p < 0.01,

** p < 0.05,

* p < 0.1. Robust standard errors for clustering at the district and county levels are in parentheses. All estimates control for individual and time fixed effects, as well as control variables of individual, occupational, and regional characteristics.

The results in Table 11 show that there is a significant urban-rural difference in the effect of COVID-19, with rural areas being affected much more than urban areas, and men being affected more negatively.

### Telecommuting experience

Under the influence of COVID-19, there have been many changes in the way people work. The rapid spread of telecommuting is the most noticeable of these changes. This section explores the impact of telecommuting on income under COVID-19 and the gender differences.

CFPS2020 asked respondents about their work patterns in February and March 2020, and categorized the answers by frequency of telecommuting use as fully using, mostly using, occasionally using, and not using. In this paper, the sample with the first three options was classified as the sample who had telecommuting experience, and the individuals who did not use telecommuting were set as the sample who had no telecommuting experience. After excluding missing values, the sample size in 2020 is 3550, and the total sample size of panel data is 11,335.

Table 12 describes the data on telecommuting experience, which shows that a larger proportion of the sample had no telecommuting experience overall, and a larger proportion of women had telecommuting experience compared to men.

**Table 12:** Data description of telecommuting experience.

	**Whole sample**		**Male**		**Female**	
Telecommuting experience	YES	NO	YES	NO	YES	NO
Observations	1,566	1,984	870	1,230	696	754
Proportion (%)	44.113	55.887	41.429	58.571	48	52

The sample was divided by telecommuting experience and regressed on equation (6) using the same method as the baseline regression.

The results in Table 13 indicate that telecommuting experience significantly mitigates the negative shock from COVID-19, overall. It may because workers who cannot work at home suffered greater loss of income. In addition, as Garrote et al. (21, 36) found out, workers who earned more before COVID-19 are more likely to be able to work from home. The results are consistent with their views. However, the comparison between genders shows that telecommuting experience has a less mitigating effect on men's income, while it is highly evident in the female group. It may because telecommuting brings the balance of work and life, decreasing the pressure on housework and childcare for women, as in the conclusions of Del Boca et al. (19, 37, 38). In addition, for the group without telecommuting experience, there was little difference between men and women affected by COVID-19. However, in the group with telecommuting experience, men were significantly negatively affected, while women showed a tendency to increase their income, although this tendency was not significant. Therefore, it can be concluded that telecommuting experience is an important mechanism for gender differences in the impact of COVID-19.

**Table 13:** Impact of telecommuting experience.

	**Who had telecommuting experience**			**Who had no telecommuting experience**		
	**Whole sample**	**Male**	**Female**	**Whole sample**	**Male**	**Female**
COVID-19 shock	−7.329^***^	−13.14^***^	0.543	−13.60^***^	−13.63^***^	−12.80^**^
	(2.720)	(3.235)	(4.966)	(3.980)	(4.375)	(5.998)
Observations	4,929	2,775	2,154	6,406	4,026	2,380
	0.558	0.568	0.547	0.551	0.527	0.598

*** *p* < 0.01,

** *p* < 0.05, * *p* < 0.1. Robust standard errors for clustering at the district and county levels are in parentheses. All estimates control for individual and time fixed effects, as well as control variables of individual, occupational, and regional characteristics.

## Conclusion and policy implications

As a catastrophe that has not occurred in a hundred years in human history, COVID-19 has had a profound impact on people's lives, and the labor market has also been greatly affected. The impact of COVID-19 on income concerns every worker, and its gender difference will also affect the income distribution pattern of the whole labor market. It is important to investigate the impact of COVID-19 on income and its gender gap, which can help to protect people's income and reduce the gender wage gap in the post-COVID-19 era.

Based on the panel data of CFPS 2014–2020, this paper analyzes the impact of COVID-19 on income and its gender differences using the Generalized DID method, and explores the mechanism of telecommuting. The following main conclusions were reached after a rigorous test: 1. COVID-19 has a significant negative impact on residents' income, and men are more negatively affected than women 2. Telecommuting can mitigate the income loss caused by COVID-19, and telecommuting is an important factor that causes women to be less affected by COVID-19 than men. 3. COVID-19 has a significant negative impact on the income of those with low education, but not on those with high education. 4. Men working in production and transportation, as well as female workers in commerce and services, suffer the greatest loss of income. 5. As men get older, their income is more affected by the epidemic, while women have the opposite trend. 6. COVID-19 has a much greater negative impact on the income of rural residents than urban residents.

Based on the findings above, this paper proposes the following recommendations for raising the income level and narrowing the gender wage gap in China in the post-epidemic era:

First, the relationship between COVID-19 prevention measures and economic development should be carefully considered. In the face of COVID-19, safety has always been the top priority, but economic development and residents' income are related to people's livelihood and cannot be ignored. How to reduce the economic losses caused by COVID-19 on the premise of ensuring the safety of residents' lives is the key and difficult point of policy formulation in the post-COVID-19 era. With the continuous development of COVID-19, governments at all levels should view COVID-19 from a dialectical perspective in the process of formulating epidemic prevention policies, and have a full understanding of the transmission characteristics and hazards of the COVID-19 virus at each stage. Under the premise of the supremacy of the people, the formulation of policies should be based on the circumstances. At the same time, it is necessary to guide the public to understand the virus, and actively promote protective measures such as vaccination and mask wearing. Under the condition that the development of COVID-19 is controllable, the resumption of work and production should be accurately promoted, and the safety inspection and emergency plan should be prepared in advance, so that the sudden localized COVID-19 can be detected, checked, and dealt with quickly to avoid the spread of COVID-19.

Second, telecommuting should be promoted. Telecommuting has played an important role during COVID-19, greatly mitigating the negative impact of COVID-19, while also accelerating the development of some industries and occupations. For industries that can better adapt to telecommuting, such as the Internet and media industries, the willingness of enterprises to work remotely should be enhanced and the rights and interests of telecommuters should be protected. For industries with difficulties with telecommuting, such as catering and domestic services, it should be ensured that they can work in a timely manner when the risk of COVID-19 is low. In addition, it is necessary to accelerate the digital transformation of the industry, improve the flexibility of telecommuting in terms of operation, management, service, etc., as well as the feasibility of operating with an Internet platform, to alleviate the impact of COVID-19. However, it should be noted that the literature review section has pointed out that the average income of industries with strong remote working adaptability is generally higher, so the latter has greater significance for economic development and income distribution.

Third, the vulnerable groups should be protected. It is found above that the impact of COVID-19 on different groups is quite different, and the groups that are already vulnerable tend to be more affected, which is bound to increase income inequality and is not conducive to the realization of the strategic goal of common prosperity. Therefore, more attention needs to be paid to vulnerable groups, and multiple measures should be taken to ensure their income. First of all, we should speed up the improvement of the basic education system, strictly implement the nine-year compulsory education, and extend the compulsory education to the high school level when the conditions are appropriate, so as to improve the overall education level of the people. Secondly, for practitioners in industries more severely affected by COVID-19, adaptive training should be provided to help them adapt to industry transformation. For practitioners in industries with difficulties in digital transformation, green channels should be opened for them during the COVID-19 period according to necessity, and COVID-19 prevention measures should be strictly implemented. In the case of ensuring the basic supply of the industry, the practitioners should be appropriately diverted, some training for career change should be provided, and appropriate subsidies should be given if necessary. Finally, it is necessary to accelerate the integration of urban and rural areas and promote the realization of the strategic goals of rural revitalization.

## Data availability statement

Publicly available datasets were analyzed in this study. This data can be found here: http://www.isss.pku.edu.cn/cfps/index.htm.

## Author contributions

The author confirms being the sole contributor of this work and has approved it for publication.

## Conflict of interest

The author declares that the research was conducted in the absence of any commercial or financial relationships that could be construed as a potential conflict of interest.

## Publisher's note

All claims expressed in this article are solely those of the authors and do not necessarily represent those of their affiliated organizations, or those of the publisher, the editors and the reviewers. Any product that may be evaluated in this article, or claim that may be made by its manufacturer, is not guaranteed or endorsed by the publisher.

## Footnotes

1 ➀ Global Confirmed. *Johns Hopkins University & Medicine*. Available online at: https://coronavirus.jhu.edu/ (accessed August 28, 2022).

2 *World Bank Open Data*. Available online at: https://data.worldbank.org/ (accessed August 28, 2022).

3 I picked up the respondents who were interviewed at least twice, and the data for 2020 must be included.

4 On January 23, 2020, the Wuhan City Novel Coronavirus Prevention and Control Command Center issued Notice No.1 declaring Wuhan was put on lockdown. In addition, the first case of Novel Coronavirus was found in most areas at the end of January, followed by a nationwide outbreak of COVID-19. Therefore, in this paper, February 2020 is taken as the initial month of COVID-19 impact, and the previous months are considered as the regular months before COVID-19.

5 The surveyed months for the sample in CFPS2020 were from July to December 2020.

6 According to Lechner et al. (39), the FE estimators of the DID model may deviate when using unbalanced panel data. So, I estimate the model using balanced data, and the results are basically the same as the baseline regression, they are not shown due to space limitation.

7 The number of months affected by COVID-19 was calculated as the number of months from February 2020 to the month prior to the month in which the respondent was interviewed in which there were new cases present in his or her province.

8 One sample with missing ethnic variables was removed.
